# Incidence, reasons, and risk factors for 30-day readmission after lumbar spine surgery for degenerative spinal disease

**DOI:** 10.1038/s41598-020-69732-2

**Published:** 2020-07-29

**Authors:** Pyung Goo Cho, Tae Hyun Kim, Hana Lee, Gyu Yeul Ji, Sang Hyuk Park, Dong Ah Shin

**Affiliations:** 1 Department of Neurosurgery, Ajou University College of Medicine, Suwon, Republic of Korea; 2 Graduate School of Public Health, Yonsei University, Seoul, Republic of Korea; 3 Department of Neurosurgery, Yonsei University College of Medicine, 50 Yonsei-ro, Seodaemun-gu, Seoul, 120-752 Republic of Korea; 4 Department of Neurosurgery, Seoul Now Hospital, Seongnam, Republic of Korea

**Keywords:** Outcomes research, Risk factors

## Abstract

This study investigated risk factors for 30-day readmission of discharged patients who had undergone lumbar spinal surgery. This retrospective, case–control study reviewed 3,933 patients discharged after elective spinal surgery for lumbar degenerative diseases from 2005 to 2012 at a university hospital. Of these patients, 102 were re-hospitalized within 30 days of discharge. Patient medical records were reviewed. The incidence of readmission within 30 days was 2.6%, and uncontrolled pain was the most common reason for readmission. In the univariate analysis, age, mental illness, the number of medical comorbidities, previous spinal surgery, fusion surgery, number of fusion levels, estimated blood loss, operation time, intensive care unit (ICU) admission, length of hospital stays, and total medical expenses were associated with a higher risk of readmission within 30 days. Multiple logistic regression analysis revealed that previous spinal surgery, operation time, ICU admission, length of hospital stays, and total medical expenses were independent risk factors for 30-day readmission. Independent risk factors for readmission were longer operation time, a previous spinal surgery, ICU admission, longer hospital stays, and higher medical expenses. Further studies controlling these risk factors could contribute to reducing readmission and thus improving the quality of care.

## Introduction

Surgical treatment of the spine has increased rapidly in recent decades and has been accompanied by increasing costs.^1^ The growth rate of spinal surgery is higher than expected, even after adjusting for the aging population.^2,3^ There is growing interest in advanced technology and equipment, which allow for more complex spinal surgeries but also lead to additional complications and costs.^4^ Unfortunately, the higher rate of spinal surgery is also associated with lower patient satisfaction.^5^

There is increasing awareness that management strategies are needed to improve the results of spine surgery while controlling costs. Lack of management of the quality of healthcare during treatment increases unplanned risks.^6^ One helpful indicator of healthcare quality is readmission within 30 days of surgery.^7,8^ Readmission under certain circumstances is related to errors in initial treatment. As such, early readmission can be used as an indicator of treatment quality.^7,9,10^

Previous studies have examined readmission rates after spinal surgery^6,7,10–22^ However, few studies in this area have focused on patients in Asian countries, such as the Republic of Korea. Racial differences are known to exist in 30-day readmission rates after spinal surgery^23^ If risk factors for readmission rates can be determined, including demographic, clinical, and therapeutic profiles, clinicians may be able to reduce readmission rates. To this end, the present study investigated the risk factors for readmission of discharged patients who underwent lumbar spinal surgery.

## Methods

### Subjects

This was a retrospective analysis of data collected between Jan. 2005 and Dec. 2012 at a single neurosurgery department in a 2,266-bed tertiary hospital with six spine surgeons. Data were obtained using private surgical data base of spine center in Severance hospital. Institutional Review Board approval of Severance Hospital, Yonsei University College of Medicine, was obtained before study initiation (Approval number: 4-2013-0605).

All variables were investigated through patient medical records, a private surgical database, and telephone interviews. Due to the retrospective nature of the study, the Institutional Review Board has confirmed that prior informed consent is not required. Patients were included in the study if they underwent elective spine surgery for lumbar degenerative disease and experienced unplanned readmission within 30 days of discharge. Lumbar degenerative disease included herniated disc disease, degenerative disc disease, spinal stenosis, spondylolisthesis, or spinal instability. The readmission group was captured using the computational algorithm that extracts all patients with 30-day readmission after an index spine surgery. In addition, telephone interviews were conducted to identify cases of readmission to other hospitals. A total of 9,587 patients underwent spine surgery at our institute during the study period. Some patients underwent multiple operations at the same time; however, they were counted as one. Among them, 3,933 patients underwent elective lumbar spine surgery, and 102 (2.6%) patients had unplanned readmission within 30 days of discharge after the initial operation. The control group (n = 487) was sampled by the propensity score matching using the 3 variables: sex, insurance type, and type of degenerative disease, with a ratio of 5 controls to 1 case. Patients were excluded if they underwent planned readmission for a second treatment, or another surgery not associated with the first surgery. Planned anti-cancer therapy and planned second surgeries were categorized as planned readmission and were excluded from the data. A total of 102 hospitalized patients were matched 1: 5 and initially 510 were extracted. However, 23 patients with data problem were excluded from the control group.

### Variables

The independent variables evaluated in this study were selected on the basis of previous research.^6,24,25^ The causes of readmission were categorized as surgical reasons directly related to the initial surgery and non-surgical reasons indirectly related to the initial surgery. The variables were categorized according to demographic, clinical, and therapeutic profiles. Demographic data including sex, age at surgery, type of insurance, marital status, educational level, and residential area were analyzed. Insurance type was categorized as health insurance, medical aid, or other. Residential area was categorized as the same city as our institute (Seoul), a neighboring city that shared a border with Seoul, or another, more distant city. Clinical profiles including comorbidities, previous spinal surgery, mental illness, and the number of comorbidities were analyzed. Comorbidities included hypertension, diabetes, hepatitis, pulmonary tuberculosis, coronary artery disease, and other. Mental illness included depression, anxiety disorder, somatoform disorder, and other disorders diagnosed by psychiatrists. There was a maximum of five comorbidities per patient. The number of comorbidities was categorized from 0 to 3 + and analyzed as categorical variables. For therapeutic profiles, the type of surgery, level of surgery, operative level, blood loss, operation time, medical expense, length of stays in the hospital, admission to the intensive care unit (ICU), and surgeon experience were analyzed. Surgical approaches were categorized as anterior, posterior, lateral, and mixed.

### Statistical analysis

Parametric data were expressed as mean ± standard deviation and were compared using Student *t*-tests. Nonparametric data were expressed as medians and compared using Mann–Whitney U tests. Chi-Square tests were used to analyze categorical variables. To identify factors for readmission, we performed multiple logistic regression analysis, which included all previously analyzed variables that were statistically significant. We examined the value of the variation inflation factor (VIF) to see if there was multicollinearity between the variables. The VIF analysis did not include adverse events (AE). And only variables with the VIF less than 10 were used in this study. We used SAS software for Windows (SAS Institute Inc., Cary, NC) for the statistical analyses. *P* < 0.05 was considered statistically significant.

## Results

### Reasons for readmission

**Figure 1:**
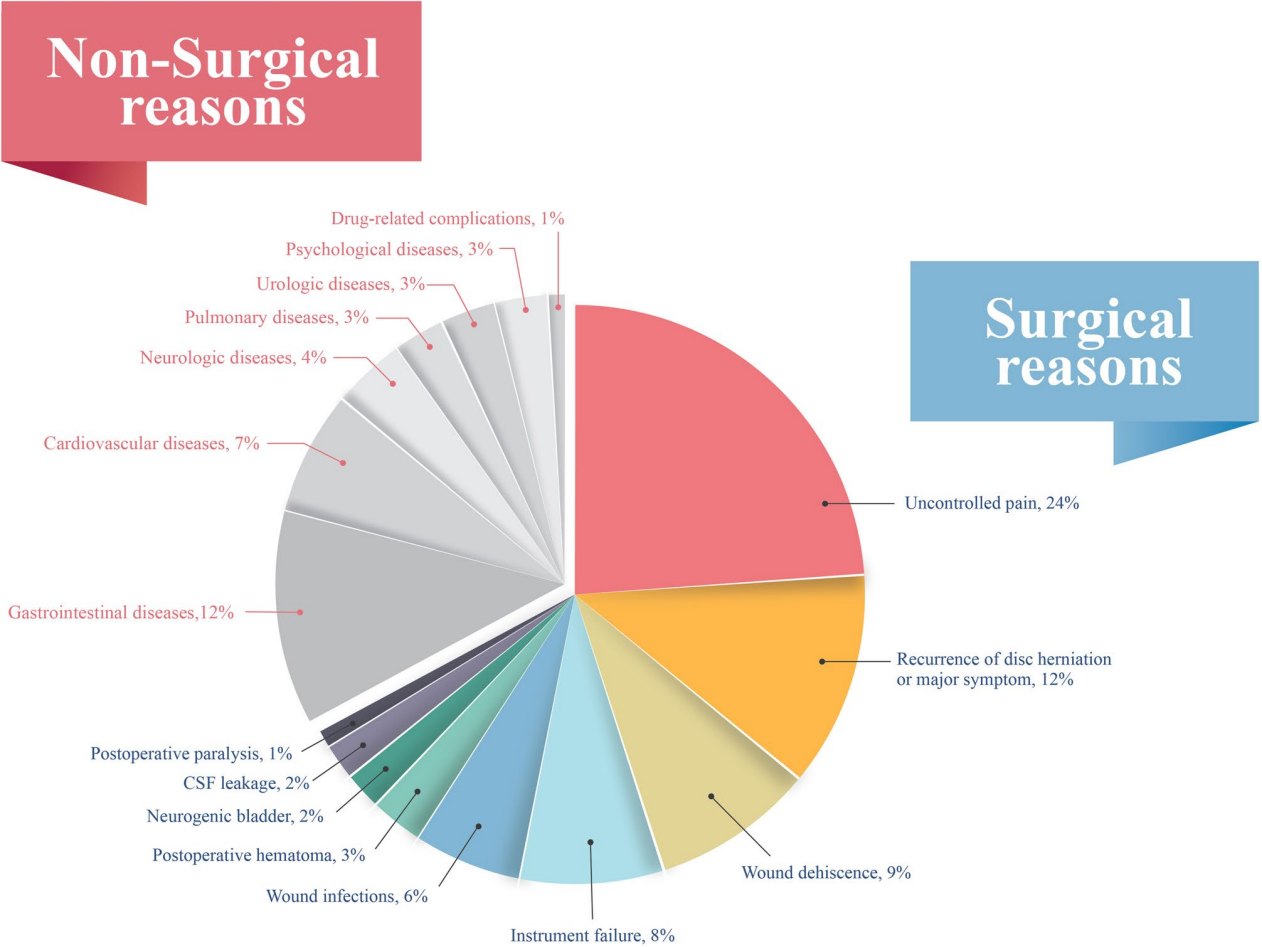
The causes of readmission after lumbar spinal surgery.

Surgical reasons for readmission included uncontrolled pain (24%), recurrence of disc herniation or major symptoms (12%), wound dehiscence (9%), instrument failure (8%), wound infections (6%), postoperative hematoma (3%), neurogenic bladder (2%), cerebrospinal fluid (CSF) leakage (2%), and postoperative paralysis (1%). Non-surgical reasons included gastrointestinal diseases (12%), cardiovascular diseases (7%), neurologic diseases (4%), pulmonary diseases (3%), urologic diseases (3%), psychological diseases (3%), and drug-related complications (1%). (Fig. 1).

### Risk factors for readmission

In the univariate analysis, age, mental illness, the number of medical comorbidities, previous spinal surgery, fusion surgery, number of fusion levels, estimated blood loss, operation time, ICU admission, length of hospital stay, and medical expenses were associated with a higher 30-day readmission rate (Table 1).

**Table 1:** Independent risk factors for unplanned 30-day readmission related to the initial operation assessed by comparison of the readmitted group and the control group.

Category	Variable	Readmission group (n = 102), n (%)	Control group (n = 487), n (%)	*P*
**Demographic variables**				
Age	Years	59.3 ± 15.1	54.7 ± 14.1	< 0.001*
Sex	Male	52 (51.0)	250 (51.3)	0.948
	Female	50 (49.0)	237 (48.7)	
Marital status	Married	96 (94)	444 (91.2)	0.413
	Single	6 (6)	43 (8.8)	
Education	University or higher	27 (26.5)	179 (36.8)	0.387
	High school	48 (47)	194 (39.8)	
	Junior high or lower	27 (26.5)	114 (23.4)	
Insurance type	Health insurance	92 (90.1)	464 (95.3)	0.135
	Medical aid	6 (5.9)	14 (2.9)	
	Other	1 (1.0)	9 (1.8)	
Residence	Seoul	57 (55.9)	209 (42.9)	0.595
	Neighboring city	25 (24.5)	144 (29.6)	
	Other city	20 (19.6)	134 (27.5)	
**Clinical variables**				
Mental illness		12 (11.8)	12 (2.5)	< 0.001*
Number of medical co-morbidities	0	30 (29.4)	281 (47.7)	< 0.001*
	1	36 (35.3)	215 (36.5)	
	2	26 (25.5)	73 (12.4)	
	> 3	10 (9.9)	20 (3.4)	
Previous spinal surgery		26 (25.5)	61 (12.15)	< 0.001*
**Therapeutic variables**				
Non-fusion surgery		33 (32.4)	220 (45.2)	0.017*
Fusion surgery		69 (67.6)	267 (54.8)	0.017*
Number of fusion levels		1.5 ± 1.7	1.0 ± 1.2	< 0.001*
Surgical approach	Anterior	23 (22.6)	142 (29.2)	0.205
	Posterior	76 (74.5)	337 (69.2)	
	Lateral	0 (0)	4 (0.8)	
	Mixed	3 (2.9)	4 (0.8)	
Blood loss (cc)		898.8 ± 1572.7	432.1 ± 653.5	< 0.001*
Operation time (min)		263.4 ± 118.8	203.0 ± 86.8	< 0.001*
ICU admission		22 (21.6)	11 (2.3)	< 0.001*
Length of hospital stays		16.4 ± 14.7	12.7 ± 13.4	0.013*
Medical expenses (won)		11,660,000 ± 6,784,400	4,463,307 ± 2,377,078	< 0.001*
Surgeon experience	Years	12.1 ± 5.8	12.6 ± 5.6	0.334

*Statistically significant (*P* < 0.05).

However, multiple logistic regression analysis showed that operation time (Odds ratio [OR] 1.595, 95% confidence interval [CI] 1.591–2.054, *P* = 0.046), previous spinal surgery (OR 2.519, 95% CI 1.075–5.094, *P* = 0.033), ICU admission (OR 1.935, 95% CI 1.753–2.425, *P* = 0.008), length of hospital stays (OR 1.447, 95% CI 1.412–1.920, *P* = 0.004), and medical expenses (OR 1.470, 95% CI 1.423–1.879, *P* = 0.001) were independent predictors of readmission within 30 days (Table 2). Risk factors with odds ratio above 2.0 is previous spinal surgery. Risk factors with odds ratios between 1.0 and 2.0 are: operation time, ICU admission, length of hospital stays, and medical expenses.

**Table 2:** Independent risk factors for unplanned 30-day readmission assessed by multiple logistic regression analysis.

Category	Variable	*P*	Odds ratio	95% CI low	95% CI high
Demographic variables	Age	0.537	0.990	0.958	1.022
Clinical variables	Mental illness	0.386	0.528	0.528	7.188
	Number of medical co-morbidities	0.198	1.310	0.871	1.970
	Previous spinal surgery	0.033*	2.519	1.075	5.094
Therapeutic variables	Non-fusion surgery	0.358	0.262	0.214	0.562
	Fusion surgery	0.179	0.462	0.154	1.387
	Number of fusion levels	0.164	1.328	0.894	1.973
	Blood loss	0.520	1.126	1.027	1.331
	Operation time	0.046*	1.595	1.591	2.054
	ICU admission	0.008*	1.935	1.753	2.425
	Length of hospital stays	0.004*	1.447	1.412	1.920
	Medical expenses	0.001*	1.470	1.423	1.879
	Surgeon experience	0.418	0.975	0.917	1.036

*Statistically significant (*P* < 0.05).

## Discussion

This is a retrospective analysis of readmission. Previous studies have reported readmission rates after spine surgery.^6,7,10–22^ Most of these studies were retrospective cohort studies that used public databases. Although Korea also has a public database for its National Health Insurance, which covers 98% of the overall population, the accuracy of diagnostic data is an issue because of the nature of claims data. Claims data are not gathered for clinical purposes but rather to reimburse healthcare services. It is possible that the diagnostic information in claims data are susceptible to up-coding by providers seeking higher reimbursement rates.^26^ However, in our study, well-controlled data were collected from our private registry.

This study found a 30-day readmission rate of 2.6%. Compared with data from other published studies, our 30-day readmission rate was relatively low.^6,10,12^ A longer initial hospital stays (mean 13.3 days) compare to previous studies^27–29^ (3.6–4.8 days) is considered to be the main reason for our low readmission rates, as early complications may have been detected during the initial hospital stays. In this study, 8% of patients were unable to interviewed due to phone number changing or refusal. The possibility of readmission to another hospital exists, which may be another reason for the relatively low readmission rate in this study. Among the 102 readmitted patients, 67% were readmitted for surgical reasons; 40% of these readmitted patients underwent another operation. The reasons for reoperation included recurrence (8, 30%), wound dehiscence (8, 30%), instrument failure (8, 30%), wound infection (2, 7%), and hematoma (1, 4%). In contrast to our results, previous studies have reported that non-surgical complications were the most common reasons for readmission, with only a minority of readmitted patients requiring reoperation.^30–32^ Although some previous studies have also cited infection as the most common cause for readmission,^33^ intractable pain was the most common reason for readmission in our study. Adogwa et al.^30^ also found that intractable pain was the most common reason for readmission. While early infection can be identified during the postoperative hospital stays, after discharge with routine medications of lower intensity, rebound pain with or without recurrence can be a major problem. Better education and medications given as needed might be a way to prevent readmission for intractable pain.

Among demographic variables, age was the only factor that was significantly associated with readmission in the univariate analysis. Previous studies have also identified age as a factor that increases the risk of readmission.^9,21,22,34^ Therefore, a multidisciplinary approach should be undertaken in older patients to reduce readmission and improve healthcare quality.^9,21,22^ Among clinical variables, previous spinal surgery, mental illness, and the number of comorbidities were statistically significant in the univariate analyses. Previous studies have identified comorbidities as factors that increase the rate of revision surgery or complicate revision surgery.^22^ Furthermore, patients suffering from mental illnesses had a higher risk of readmission.^25,34,35^ In the current study, a revision surgery was identified as a factor that increases the risk of readmission. Among published studies, the effect of previous spine surgery on readmission remains unclear; one study found that readmission was more common among patients with a history of additional procedures than among those without such a history,^2^ whereas another study found no relationship between a previous spine surgery and readmission.^20^

Among therapeutic profiles, medical expenses and length of hospital stays were the only variables that were significantly associated with readmission rates. Previous studies have also identified the number of fusion levels, length of hospital stays, and ICU admission as factors for readmission after spinal surgery.^20,25^ Some studies have found that the length of hospital stays increases the risk of readmission, while others have found that it reduces the risk of readmission.^25^ Identification of medical expenses as a factor for readmission is consistent with previous studies. Repeated hospitalization requires greater medical resources, leading to higher medical expenses. A previous study identified operation time, but not blood loss during surgery, as a factor for readmission,^20^ Similarly, operation time was significantly associated with readmission in this current study. Operation time is a factor for readmission, possibly because operation time is an indicator of the complexity of the surgery.^36^ Surgeons with greater experience tend to perform operations more rapidly and skillfully, so surgeon experience may be related to a shorter operation time.^37^ Surgeon, who has a lot of experience, is believed to reduce the readmission due to the reduced possibility of postoperative complication. ICU admission has been associated with increased risk of readmission in previous studies.^32^ ICU admission is an indicator of the severity of the patient's condition due to medical complications and increased surgical difficulty; thus, patients admitted to the ICU should be given special care during hospitalization.^25^

Previous studies have reported that patterns of medical care are influenced by the type of insurance and other socioeconomic factors.^38,39^ We expected that the type of insurance utilized would affect the readmission rate. However, we found no such relationship. We also expected that proximity to the hospital would be associated with a higher readmission rate, but we did not find any relationship between these two variables.

Several limitations of our study should be noted. First, this was a retrospective study performed via medical record review. Second, this study was conducted on patients who underwent spine surgery in a single hospital, so patients who were readmitted at another hospital were not accounted for. These could limit the validity of the data. Third, AE are the main cause of re-admission, however these were excluded from this study due to multicollinearity with other variables. We performed statistics on preliminary AE before conducting this study. Statistical results confirmed that multicollinearity problems occur when AE is directly included. The variables such as operation time, blood loss, length of stay and admission to ICU used in this study are factors that can be indirectly affected by AE. Because of this, not all AE have been eliminated. In future work, we will discuss the direct impact of AE on readmission.

## Conclusion

The incidence of 30-day readmission after spine surgery was 2.6%, and intractable pain was the most common reason for readmission. Multiple logistic regression analysis revealed that longer operation time, a previous spinal surgery, ICU admission, longer hospital stays, and higher medical expenses were independent risk factors for readmission. Further efforts to manage these risk factors will reduce readmission and thus improve quality of care.
